# Next-Generation Metabolic Reprogramming in iPSC-Derived Cardiomyocytes: CRISPR-EV Synergy for Precision Cardiac Regeneration

**DOI:** 10.3390/biom16030467

**Published:** 2026-03-20

**Authors:** Dhienda C. Shahannaz, Tadahisa Sugiura

**Affiliations:** 1 Digestive Disease & Surgery Institute, Cleveland Clinic, Cleveland, OH 44195, USA; dhiendaladdynasrul@gmail.com; 2 Department of Cardiothoracic and Vascular Surgery, Montefiore Medical Center, Albert Einstein College of Medicine, Bronx, NY 10461, USA

**Keywords:** iPSC-derived cardiomyocytes, CRISPR metabolic engineering, extracellular vesicles, cardiac regeneration, mitochondrial biogenesis, metabolic reprogramming, oxidative phosphorylation, cardiomyocyte maturation, EV bioengineering, metabolic biomarkers

## Abstract

Cardiovascular disease remains the leading global cause of mortality, largely due to the limited regenerative capacity of adult human myocardium. Induced pluripotent stem cell-derived cardiomyocytes (iPSC-CMs) offer a scalable platform for cardiac repair and disease modeling; however, their persistent metabolic immaturity—characterized by reliance on glycolysis, reduced oxidative phosphorylation (OXPHOS), and structurally underdeveloped mitochondria—limits functional integration and long-term therapeutic efficacy. Recent advances indicate that targeted metabolic reprogramming can enhance mitochondrial biogenesis, increase ATP production, and improve stress resilience in iPSC-CMs. This review examines the complementary integration of CRISPR-based metabolic engineering and extracellular vesicle (EV)-mediated metabolic modulation as a systems-level strategy for cardiac maturation. We discuss CRISPR activation, interference, and epigenome-editing approaches targeting regulators such as PGC-1α, TFAM, and PPARs to promote stable enhancement of mitochondrial networks and respiratory capacity. In parallel, engineered EVs delivering miRNAs, metabolic enzymes, and redox modulators provide non-genomic mechanisms to optimize bioenergetic function and mitigate oxidative stress. By synthesizing mechanistic insights, quantitative bioenergetic metrics, and translational considerations, we propose CRISPR-EV synergy as a precision framework for durable metabolic maturation of iPSC-CMs, with implications for regenerative therapy, pharmacologic screening, and myocardial repair.

## 1. Introduction

Cardiovascular disease remains the leading global cause of mortality and long-term morbidity, largely because adult mammalian myocardium exhibits very limited innate regenerative capacity after injury such as myocardial infarction or chronic ischemia [1,2,3]. Unlike lower vertebrates that can reconstitute functional myocardium via dedifferentiation and proliferation, adult human cardiomyocytes exit the cell cycle shortly after birth and manifest low proliferative potential, rendering lost myocardium into essentially permanent scar tissue [1,3,4]. The postnatal heart undergoes a metabolic maturation shift from predominant glycolysis to high-capacity fatty acid oxidation (FAO) [1,3,5], a transition associated with increased reactive oxygen species (ROS), mitochondrial expansion, and decreased cardiomyocyte proliferation [1,3,4,5,6]. Manipulating this metabolic switch has been recognized as a promising lever to unlock latent regenerative programs in adult heart tissue and human cell models alike [1,2,3,4,5,6,7,8,9,10,11,12,13,14,15,16].

Human induced pluripotent stem cell-derived cardiomyocytes (iPSC-CMs) have emerged as a fundamental platform for cardiac disease modeling, drug screening, and regenerative therapy development because they recapitulate human cardiac gene expression and electrophysiology in vitro [1,2]. However, iPSC-CMs currently retain a fetal-like metabolic phenotype characterized by reduced oxidative phosphorylation capacity, persistent reliance on glycolytic metabolism, and structurally immature mitochondria with limited fatty acid utilization [1,5,6]. This metabolic immaturity constrains their ability to model adult cardiac bioenergetics accurately and diminishes functional integration following transplantation [1,2,5,6].

Directed metabolic maturation strategies—including fatty acid supplementation and activation of peroxisome proliferator-activated receptor (PPAR) and PGC-1α signaling—have been shown to improve mitochondrial biogenesis, oxidative phosphorylation, and structural–electrical maturation [1,4,6]. Nevertheless, even optimized metabolic culture conditions fail to fully recapitulate adult cardiomyocyte energetics, and metabolically matured iPSC-CMs often display heightened vulnerability to hypoxic stress or oxidative stress, highlighting a trade-off between metabolic sophistication and stress resilience that remains unresolved.

The need for precision metabolic reprogramming in iPSC-CMs therefore arises from this central bottleneck: the absence of a modular framework capable of both overcoming fetal metabolic bias and establishing durable, adult-like mitochondrial function compatible with high ATP demand, redox stability, and substrate flexibility [4,6]. Precision metabolic reprogramming implies targeted intervention at key regulatory nodes governing cardiac metabolic networks, including transcriptional coactivators (PGC-1α, PPARs), glycolytic suppressors (HIF1α, LDHA), and regulators of mitochondrial dynamics and biogenesis. Effective reprogramming must also engage the core mitochondrial bioenergetic pathways—hallmarks distinguishing adult cardiomyocytes from their immature counterparts [1,2,3,4,5,6,7,8,9,10,11,12,13,14,15,16].

In this context, a systems-oriented framework for cardiac metabolic engineering is required to move beyond linear optimization strategies toward coordinated, multi-layer metabolic remodeling. Advances in systems biology and network medicine indicate that sustained metabolic maturation arises from synergistic modulation of transcriptional control, mitochondrial architecture, and intercellular metabolic signaling rather than from isolated single-factor interventions [2,4,16,17,18]. This perspective supports integration of high-precision genetic engineering tools, such as CRISPR/Cas-based approaches [4,6,17,19], with extracellular metabolic modulators, including engineered extracellular vesicles (EVs) [4,16,20,21], to reprogram metabolic flux at cellular and tissue scales. References [8,22] correspond to accepted manuscripts and/or preprints available on bioRxiv or publisher early-view platforms at the time of submission.

Such integrative strategies enable coordinated enhancement of oxidative metabolism, redox homeostasis, and bioenergetic adaptability in iPSC-CMs while acknowledging mechanistic constraints revealed by lineage-tracing and functional studies showing that EVs primarily exert paracrine metabolic and anti-fibrotic effects rather than directly inducing cardiomyocyte proliferation [1,4,6,16,23,24]. Accordingly, iPSC-CM maturation should be reframed, not as incremental culture optimization, but as coordinated metabolic network engineering linking molecular interventions to measurable functional cardiac outcomes.

In sum, overcoming metabolic immaturity in iPSC-CMs requires a coordinated, systems-level strategy rather than incremental optimization of culture conditions. Integrative metabolic engineering—combining targeted genetic modulation and extracellular signaling platforms within a quantitative network framework—offers a unified approach to reconfigure mitochondrial function, redox balance, and substrate utilization in a durable and programmable manner. By linking molecular interventions to measurable functional cardiac endpoints, this framework establishes a translational pathway from metabolic control to improved disease modeling, pharmacologic screening fidelity, and regenerative therapeutic development. Where applicable, quantitative ranges discussed herein reflect consensus trends across multiple independent preclinical studies rather than isolated experimental outcomes.

## 2. Metabolic Landscape of iPSC-CMs

iPSC-CM are indispensable tools for cardiac disease modeling, drug discovery, and regenerative medicine [1,2,3,4,5,6]. Yet, the metabolic profile of iPSC-CMs remains fundamentally immature, reflecting a developmental trajectory that diverges substantially from adult cardiomyocytes [6]. This chapter systematically delineates the metabolic architecture, the dynamic regulation of energy pathways, and the biomolecular signatures that define iPSC-CM metabolism, with emphasis on glycolytic bias versus oxidative phosphorylation (OXPHOS) [1,6], mitochondrial structure and dynamics [6], as well as key indicators such as mitochondrial membrane potential, NAD^+^/NADH balance, and reactive oxygen species (ROS) regulation [4,6].

### 2.1. Glycolytic Bias vs. Adult Cardiomyocyte Oxidative Phosphorylation

Adult cardiomyocytes are among the most energetically demanding cells in the human body [4,6], with ATP needs primarily met by mitochondrial OXPHOS fueled by fatty acid oxidation (FAO) and pyruvate oxidation via the tricarboxylic acid (TCA) cycle [1,4,6]. In contrast, iPSC-CMs retain a fetal-like metabolic phenotype dominated by aerobic glycolysis [1,3,6], similar to pluripotent stem cells and cancer cells (the “Warburg effect”) [1,2,12,25,26]. In mature cardiomyocytes, FAO contributes up to ~70–90% of ATP production, with glycolysis accounting for a smaller proportion [6]. Conversely, iPSC-CMs depend heavily on glycolysis for ATP even in oxygen-rich conditions, manifesting elevated expression of glycolytic regulators such as hexokinase 2 (HK2) and lactate dehydrogenase A (LDHA) [4,6,12,16], which can be >3-fold higher than in adult phenotypes [6,27]. This glycolytic reliance leads to accumulation of lactate, diminished respiratory capacity, and a 50% deficit in ATP-linked respiration compared to mature cardiomyocytes [4,6]. These fundamental bioenergetic disparities between iPSC-CMs and adult cardiomyocytes are summarized in Table 1, highlighting specific metabolic deficiencies that constitute actionable targets for precision reprogramming strategies.

Differentiation toward an adult metabolic profile can be accelerated by modulating key pathways. Chemical or RNAi-mediated inhibition of HIF1α and LDHA shifts metabolism toward OXPHOS, increases mitochondrial content, and improves structural and contractile maturation. Glycolytic suppression, coupled with stimulation of FAO and pyruvate oxidation, improves ATP generation and better mimics adult energetic states [4,6,15,16]. 

### 2.2. Mitochondrial Architecture and Dynamics

Mitochondrial structure in iPSC-CMs correlates strongly with metabolic function. Immature iPSC-CM mitochondria are typically small, spherical, perinuclear, and possess underdeveloped cristae [4], reflective of a cell prioritizing glycolysis over mitochondrial oxidation [1,4,6]. Adult cardiomyocyte mitochondria are enlarged, densely cristae-rich, and distributed throughout the sarcomere, optimizing ATP delivery for contractile function [6,36,37,38,39,40].

Mitochondrial dynamics—fusion and fission—also play critical roles in shaping metabolic competence. In pluripotent cells, mitochondria are fragmented with higher fission activity, while differentiation drives fusion, augmenting bioenergetic capacity. Fusion is regulated by mitofusins (MFN1/2) and optic atrophy 1 (OPA1), promoting a networked mitochondrial architecture that supports enhanced OXPHOS and ATP output. Fission, mediated by dynamin-related protein 1 (DRP1), facilitates quality control and metabolic flexibility. Coordinated regulation of these processes enables mitochondria to respond to energetic demands by adjusting morphology and function [6,12,13].

### 2.3. Biomolecular Signatures: Δψm, NAD^+^/NADH, and ROS

Mitochondrial membrane potential (Δψm) reflects the proton gradient that drives ATP synthesis via ATP synthase [4]. Mature cardiomyocytes exhibit robust and stable Δψm, whereas iPSC-CMs often show lower and more variable membrane potential, indicating compromised OXPHOS efficiency [4,6]. Δψm maturation correlates with increased ETC complex assembly and cardiolipin remodeling, both of which enhance electron transport efficiency [4].

The NAD^+^/NADH ratio is a central determinant of cellular energy metabolism and redox homeostasis. High NAD^+^ favors oxidative metabolism by enabling efficient electron transfer into the ETC, while elevated NADH reflects reduced electron flux and can facilitate lactate production in glycolytic states. In iPSC-CMs, NAD^+^/NADH imbalance is characteristic of glycolytic reliance and immature mitochondrial function; interventions that increase NAD^+^ availability enhance respiratory capacity and metabolic flexibility [4,6].

Reactive oxygen species (ROS) arise as byproducts of electron leakage from the ETC [4]. Moderate ROS levels act as signaling molecules, regulating gene expression, adaptation processes, and metabolic enzymes [4,6]. However, excessive ROS damages lipids, proteins, and mitochondrial DNA, impairing respiratory efficiency and contributing to cardiomyocyte dysfunction [6,41]. Adult cardiomyocytes maintain a finely tuned antioxidant system (e.g., glutathione peroxidase, superoxide dismutase) to buffer ROS [42,43,44]. In contrast, immature iPSC-CMs often lack robust ROS handling, making them more susceptible to oxidative stress during metabolic maturation [6].

### 2.4. Synthesis: A Metabolic Framework for iPSC-CM Maturation

Collectively, these features illustrate a coherent metabolic landscape in iPSC-CMs: a glycolytic dominant state with underdeveloped mitochondrial networks, subdued membrane potential, and disrupted redox balance. Transitional maturation toward adult-like OXPHOS depends on orchestrating mitochondrial biogenesis, structural remodeling, and metabolic flux regulation. This defines a systems metabolic architecture in which strategic interventions at key enzymatic and regulatory nodes can yield transformative functional gains.

Understanding this landscape with high resolution—combining transcriptomics, metabolomics, and live bioenergetic profiling—establishes a detailed blueprint for precision metabolic reprogramming strategies. By quantifying and leveraging biomarkers such as Δψm, NAD^+^/NADH ratio, and ROS dynamics, researchers can predict, monitor, and optimize the maturation trajectories of iPSC-CMs toward clinically relevant energetic phenotypes.

## 3. CRISPR-Guided Metabolic Engineering

Precision metabolic engineering holds transformative potential for advancing the maturation and functional competency of induced pluripotent stem cell-derived cardiomyocytes (iPSC-CMs). Traditional metabolic maturation strategies (chemical supplementation, substrate switching) improve mitochondrial architecture and oxidative phosphorylation (OXPHOS), but fail to fully replicate adult cardiomyocyte bioenergetics without precise modulation of key regulatory nodes. A CRISPR-guided approach enables programmable control over the expression of master metabolic regulators—such as PPARGC1A (PGC-1α), TFAM, estrogen-related receptors (ERRα/β), and peroxisome proliferator-activated receptors (PPARs)—to rewire metabolic fluxes and enhance mitochondrial respiration, ATP kinetics, and cardiac energy phenotypes [4,6].

The iPSC lines discussed in this review are primarily generated using integration-free reprogramming strategies, including Sendai virus [45], episomal plasmids [46], and mRNA-based delivery of Yamanaka factors (OCT4, SOX2, KLF4, and c-MYC) [1]. These approaches minimize genomic integration and preserve genomic stability, which is critical for downstream CRISPR-based metabolic engineering. Tissue sources most commonly include dermal fibroblasts [1] and peripheral blood mononuclear cells (PBMCs) [47], both of which exhibit residual epigenetic memory that can influence mitochondrial function, glycolytic bias, and differentiation efficiency. Variability in donor age, somatic cell origin, and reprogramming modality contributes to heterogeneity in baseline metabolic phenotypes of derived iPSC-CMs, underscoring the need for programmable and standardized metabolic modulation strategies.

### 3.1. Target Selection: PGC-1α, TFAM, ERRα/β, and PPAR Pathways

Metabolic reprogramming in cardiomyocytes revolves around coordinated transcriptional networks that control mitochondrial biogenesis, substrate utilization, and electron transport chain (ETC) assembly. Among these, PGC-1α (PPARGC1A) is a central coactivator that orchestrates downstream metabolic programs by physically interacting with nuclear receptors such as estrogen-related receptor α (ERRα) to induce mitochondrial gene expression, fusion/fission balance, and oxidative capacity. In hiPSC-CM differentiation, PGC-1α upregulation correlates with increased mitochondrial number, higher OXPHOS activity, and enhanced membrane potential (Δψm); conversely, its knockdown impairs ETC gene expression and mitochondrial respiration.

**Figure 1:**
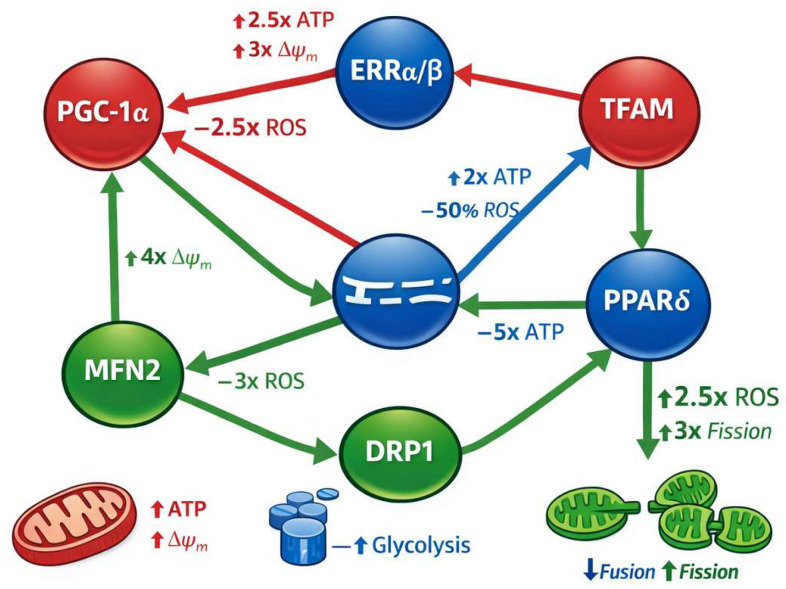
CRISPR-targetable metabolic regulatory network in iPSC-derived cardiomyocytes. Schematic representation of transcriptional regulators (PGC-1α, ERRα/β, PPARδ), mitochondrial genome controllers (TFAM), and mitochondrial dynamics proteins (MFN2, DRP1) governing oxidative phosphorylation (OXPHOS), glycolysis, and mitochondrial architecture in iPSC-CMs. Red nodes indicate OXPHOS-associated regulators, blue nodes glycolytic control, and green nodes mitochondrial fusion/fission pathways. Directed edges denote activation or inhibition effects on mitochondrial membrane potential (Δψm), ATP production, and reactive oxygen species (ROS). Quantitative annotations reflect fold-change ranges reported in recent preclinical models. In this schematic, transcriptional coactivators (PGC-1α, ERRα/β, PPARδ) are positioned upstream of mitochondrial biogenesis programs; TFAM represents mitochondrial genome control; and MFN2/DRP1 define structural dynamics that directly modulate Δψm, ATP output, and ROS balance.

As illustrated in Figure 1, PGC-1α functions as a master upstream node linking transcriptional activation to mitochondrial expansion and redox stabilization, thereby influencing ATP generation and ROS production in parallel. To contextualize these regulators within an integrated metabolic control network, Figure 1 schematically illustrates key CRISPR-addressable nodes governing mitochondrial biogenesis, substrate utilization, and redox homeostasis in iPSC-CMs [4,6].

Mitochondrial transcription factor A (TFAM) is another strategic target: it binds and packages mitochondrial DNA (mtDNA) [48,49], maintains copy number [50], and stabilizes nucleoid structure [51,52], thereby directly influencing mitochondrial genome transcription and ATP production [53]. TFAM modulation has been linked to reduced ROS generation and improved calcium handling in cardiomyocyte systems [54], underscoring its role in bioenergetic homeostasis. Within the network depicted in Figure 1, TFAM represents the principal mitochondrial genome regulatory node, translating transcriptional signals into quantitative increases in mtDNA copy number, ETC assembly, and oxidative capacity.

Mitochondrial fusion regulator MFN2 represents a critical yet often under-discussed determinant of cardiomyocyte metabolic maturation. MFN2 promotes mitochondrial elongation, cristae organization, and efficient electron transport chain coupling, thereby enhancing ATP synthesis and reducing electron leakage-driven ROS production. In cardiomyocytes, MFN2 deficiency disrupts mitochondrial distribution along sarcomeres and impairs calcium-mitochondria crosstalk, leading to compromised excitation–contraction coupling. CRISPR-mediated upregulation of MFN2 has been shown to increase mitochondrial network connectivity, stabilize Δψm, and synergize with PGC-1α-driven biogenesis programs to promote adult-like oxidative metabolism. Importantly, as shown in Figure 1 (green fusion/fission module), MFN2 operates in functional opposition to DRP1-mediated fission, and balanced modulation of these dynamics determines mitochondrial morphology, ROS buffering capacity, and bioenergetic efficiency. Accordingly, MFN2 is included in Figure 1 as a CRISPR-addressable node linking mitochondrial architecture to functional bioenergetic outcomes.

The PPAR family (PPARα, PPARδ/β) regulates fatty acid oxidation (FAO) and metabolic flexibility [6,55]. Activation of PPARδ significantly increases FAO flux, augments mitochondrial surface area, and redistributes mitochondria to energy-demand regions in cardiomyocytes [6,56,57]—hallmarks of metabolic maturation. In Figure 1, PPARδ is positioned within the OXPHOS regulatory axis (red module), reflecting its role in substrate switching from glycolysis toward fatty acid oxidation and its downstream influence on ATP production and mitochondrial membrane potential.

Collectively, these targets represent nodal points in metabolic control networks that offer leverage for CRISPR-mediated regulation to drive adult-like metabolic programs in iPSC-CMs.

### 3.2. CRISPRa/i Strategies and Base Editing

CRISPR technologies extend beyond gene knockout. CRISPR activation (CRISPRa) and interference (CRISPRi) enable programmable transcriptional modulation without altering genomic sequences. CRISPRa uses catalytically dead Cas9 (dCas9) fused to transcriptional activators (e.g., VPR) to upregulate endogenous gene expression, while CRISPRi uses dCas9 linked to repressors to downregulate undesired pathways such as glycolytic bias in iPSC-CMs [1,2,3,4,5,6,58,59]. However, CRISPR effector expression may be silenced after differentiation if integrated improperly, necessitating careful design of promoters and safe-harbor loci to ensure persistent control in cardiomyocytes.

Base editing represents another advanced modality that induces precise, programmable single-nucleotide changes without double-strand breaks, minimizing genotoxic stress. For metabolic engineering, base editors can introduce stable alterations to regulatory elements or coding regions of PGC-1α, ERRα/β, TFAM, or PPAR promoters/enhancers to enhance metabolic gene expression [57,58]. Emerging base-editing platforms have achieved high on-target efficiency with reduced off-target effects, a critical feature for translational applications.

CRISPR systems may also be combined with epigenome editing (e.g., dCas9 fused to chromatin modifiers) [60,61,62,63] to induce heritable, reversible shifts in metabolic gene networks without permanent DNA alterations.

### 3.3. Quantitative Outcomes: Respiration and ATP Kinetics

Targeted CRISPR regulation of metabolic genes produces quantifiable enhancements in mitochondrial function. Upregulation of PGC-1α and downstream effectors increases basal and maximal oxygen consumption rates (OCRs)—a direct readout of OXPHOS—and elevates ATP production beyond levels achievable with metabolic supplementation alone. Mitochondrial content and cristae density also increase, improving ETC efficiency, electron flux, and ATP synthesis rates. These CRISPR-engineered improvements have been measured as significant fold increases in respiratory metrics and ATP kinetics relative to control iPSC-CMs.

Moreover, CRISPRa-mediated enhancement of TFAM expression increases mtDNA copy number and stabilizes ETC components, further bolstering ATP output and lowering ROS production, thereby improving metabolic resilience of engineered cardiomyocytes.

### 3.4. Safety and Off-Target Considerations

Despite its precision, CRISPR technology carries inherent safety concerns. Off-target genome editing—unintended edits at loci with sequence similarity—remains a major challenge [64,65,66]. Off-target Cas9 activity can result in unpredictable genetic changes, particularly when using nucleolytic CRISPR/Cas9 systems, making comprehensive in silico prediction and experimental validation essential.

High-fidelity Cas9 variants, paired nickases, and improved-guide RNA design can reduce off-target effects [67,68,69]. Furthermore, transient delivery methods (e.g., ribonucleoprotein complexes) limit prolonged exposure to editing machinery and minimize collateral edits [70,71,72,73,74]. Epigenome editing and base editing modalities further circumvent permanent double-strand breaks, reducing genotoxic risk [75,76].

Finally, regulatory and ethical considerations for clinical translation demand rigorous characterization of CRISPR-engineered iPSC-CMs over extended periods, including genomic stability, tumorigenicity, and long-term metabolic phenotypes [10,77,78,79].

### 3.5. Synthesis and Prospects

CRISPR-guided metabolic engineering represents a systematic, high-resolution approach to overcoming iPSC-CM metabolic immaturity. By harnessing precise activation or repression of key regulatory networks—including PGC-1α, TFAM, ERRα/β, and PPAR pathways—researchers can program metabolic architectures that mimic adult cardiomyocyte bioenergetics with quantifiable gains in OXPHOS and ATP production. Continued refinement of CRISPR modalities, off-target mitigation strategies, and integration with functional assays will be crucial for realizing the translational potential of engineered cardiomyocytes in cardiac regenerative medicine.

## 4. Extracellular Vesicle Metabolic Modulators

Extracellular vesicles (EVs) function as endogenous nanoscale carriers of metabolic information—transferring lipids, proteins, nucleic acids, and metabolites between cells and thereby shaping recipient cell bioenergetics, substrate utilization, and stress responses. Unlike conventional therapeutic agents, EVs intrinsically encode metabolic cues that reflect the physiological or pathological status of their cell of origin, making them powerful agents for metabolic modulation in regenerative cardiology and systemic metabolic reprogramming.

### 4.1. EV Biogenesis and Metabolic Cargo

EVs arise through multiple cellular pathways, most notably via multivesicular body (MVB) exocytosis (producing exosomes) and plasma membrane budding (yielding microvesicles) [80,81]. During biogenesis, the parent cell selectively packages cargo—including microRNAs (miRNAs), long non-coding RNAs, metabolic enzymes, mitochondrial proteins, lipids, metabolites, and ATP-related factors—into the lumen or membrane of EVs [82,83,84]. The molecular composition of EVs closely reflects the metabolic state of the donor cell, such that stressed or energetically reprogrammed cells secrete EVs enriched in specific cargo that influence energy pathways in recipient cells.

Biomolecular sorting mechanisms involve endosomal sorting complexes required for transport (ESCRT) and lipid-driven pathways (e.g., ceramide-dependent budding), which influence cargo selection and vesicle release dynamics [85,86]. These processes determine not only EV composition but also targeting specificity, uptake efficiency, and downstream metabolic effects.

EV cargo extends far beyond nucleic acids; lipids such as ceramides and cholesterol modulate membrane fluidity and signaling cascades in recipient cells, while metabolites like glycerol and amino acids influence basal metabolic flux [86,87]. Proteins—including heat shock proteins and enzymes—can directly alter recipient cell metabolic enzyme activity and organelle function upon uptake.

### 4.2. Engineered EVs: miRNAs, Metabolic Enzymes, and Lipid Modulators

Engineering EVs enhance their therapeutic potential by tailoring cargo composition and delivery efficiency. EVs can be modified to carry specific miRNAs known to regulate cardiomyocyte metabolism (e.g., miR-199a for proliferation and glycolysis adjustment [86,87], miR-202-5p for stress resilience [14,88]), metabolic enzymes, or regulatory factors involved in fatty acid oxidation and mitochondrial dynamics.

Cardiac stromal cell-derived EVs with unique miRNA signatures (e.g., miR-1260a, miR-202-5p, and miR-451a) enhance cardiomyocyte resistance to hypoxic injury by activating survival pathways and modulating metabolic gene networks [14,89].

Beyond nucleic acids, engineered EVs can deliver mitochondrial proteins (e.g., TOM20, ATP5A1) [4,6,10,11,12,13,14,15,16] or even entire mitochondrial fragments that integrate into recipient organelle networks and restore oxidative metabolism. Mitochondria-rich EVs from iPSC-CMs have been demonstrated to increase intracellular ATP production, restore mitochondrial membrane potential, and improve contractile function in injured cardiomyocytes.

Lipid cargo—such as ceramides or sphingolipids—also affects recipient cell metabolic signaling, influencing processes like apoptosis, oxidative stress signaling, and membrane-associated enzyme activity [90]. Rational engineering of lipid components can therefore optimize EV uptake and metabolic impact. The diversity of engineered EV cargo and its corresponding metabolic effects in cardiomyocyte systems are quantitatively summarized in Table 2

### 4.3. Functional Effects in Recipient Cells

Upon internalization via endocytosis, membrane fusion, or tunneling nanotubes, EVs release cargo that can reprogram recipient cell metabolism at multiple levels:

- Bioenergetic restoration: EV-encoded mitochondrial proteins and metabolic enzymes enhance oxidative phosphorylation efficiency, increase ATP output, and improve mitochondrial dynamics through regulation of fusion (MFN1/2) and fission (DRP1) pathways.
  Redox modulation: miRNAs and enzymes carried by EVs regulate NAD^+^/NADH balance and ROS production, crucial determinants of metabolic flux and oxidative stress resilience.
  Stress adaptation: Cardioprotective miRNAs delivered by EVs can suppress pro-apoptotic signaling and subclinical metabolic dysregulation in hypoxic or post-ischemic environments.
- Substrate flexibility: EV cargo may influence glycolytic and fatty acid oxidation pathways, shifting metabolic preference toward adult-like OXPHOS patterns in immature cardiomyocytes.

Collectively, these effects translate into improved cellular survival, enhanced contractile profiles, and structural recovery in preclinical cardiac models, showcasing the broad therapeutic utility of EV metabolic modulators.

### 4.4. Dose and Delivery Optimization

Achieving optimal EV dosing and delivery is critical for maximizing therapeutic efficacy while minimizing off-target effects. Preclinical models show that EV dosing strategies must be tailored to particle count, cargo potency, and target tissue environment. For instance, mitochondria-rich EV treatment at ~1.0 × 10^8^ EV/mL was sufficient to rapidly restore ATP production in hypoxic cardiomyocytes within hours in vitro and enhance in vivo myocardial function after injury.

Key considerations for dose optimization include the following:

- Particle quantification versus cargo functionality: Measuring both particle number and biologically active cargo (e.g., miRNA copies, enzyme activity) ensures consistent metabolic impact.
- Targeted delivery: Surface modification of EVs (e.g., integrin peptides, antibodies) improves tissue specificity and uptake efficiency. Engineering EV membranes can enhance biodistribution and reduce off-target uptake.
- Route of administration: Intramyocardial, intracoronary, or systemic delivery modalities affect EV retention, half-life, and metabolic impact, necessitating comparative optimization studies.

### 4.5. Synthesis

Extracellular vesicles function as sophisticated metabolic nanocarriers, naturally integrating intercellular communication with metabolic regulation. Engineered EV platforms extend this capability, enabling precise transfer of miRNAs, enzymes, organelle components, and lipids to reshape recipient cell energy metabolism. As understanding of EV biogenesis, cargo dynamics, and dose–delivery kinetics advances, engineered metabolic modulators offer a versatile and scalable approach for precision metabolic reprogramming in cardiomyocyte regeneration and systemic energetics modulation—bridging gaps between molecular control, cellular function, and translational regenerative strategies.

While alternative approaches exist—including miRNA-mediated regulation, antisense oligonucleotides, and conventional gene knockouts—CRISPR offers distinct advantages for iPSC metabolic engineering: high-precision, programmable activation/repression, multiplexed targeting of multiple metabolic nodes simultaneously, and the potential for heritable yet reversible modulation of endogenous gene networks. Compared with RNAi or antisense approaches, CRISPR enables stable modulation without exogenous overexpression, reducing variability and allowing fine-tuned control over metabolic flux in differentiating cardiomyocytes. Limitations include off-target effects and delivery challenges, which are actively mitigated by high-fidelity nucleases, base editors, and epigenome-targeting platforms [40,41,42,47]. CRISPR/Cas systems are particularly advantageous for iPSC engineering because they allow high-efficiency, site-specific editing of endogenous loci to create isogenic lines, enable multiplexed modulation of multiple metabolic and transcriptional nodes simultaneously, and support reversible CRISPRi/CRISPRa-based transcriptional control without exogenous overexpression. This precision facilitates reproducible differentiation outcomes, minimizes clonal variability, and allows complex reprogramming of metabolic networks—capabilities that traditional miRNA, antisense, or conventional gene knockout approaches cannot achieve as efficiently [11,18,19,63].

EVs are uniquely suited for iPSC metabolic engineering compared with synthetic nanoparticle carriers due to their intrinsic biocompatibility, ability to deliver multi-component cargo (RNA, proteins, lipids, metabolites), cell-targeting specificity, and capacity to integrate into intercellular signaling networks [13,20,55,60]. Limitations include variable yield, heterogeneity, and the need for standardized cargo loading protocols, which ongoing engineering efforts are addressing [53,54,55].

Compared with synthetic lipid nanoparticles or polymer-based delivery systems, EVs generally exhibit lower immunogenicity and reduced complement activation due to their endogenous membrane composition and native surface proteins, thereby improving cellular tolerability in sensitive systems such as iPSC-CM [12,20,82,93,95,96]. However, this biological origin also introduces donor-dependent variability that can compromise batch-to-batch reproducibility [21,81,97,98]. In contrast, synthetic nanoparticles offer superior physicochemical uniformity, scalable manufacturing pipelines, and precise control over size distribution and composition, but often require surface modification to reduce immunogenicity and enhance targeting specificity [82,85,99,100]. Cargo loading efficiency likewise presents tradeoffs: nanoparticle systems frequently achieve higher encapsulation efficiency for defined nucleic acids, whereas EV cargo incorporation can be less predictable and dependent on donor cell biology or post-isolation loading methods [12,13,73,82]. From a translational standpoint, large-scale EV production remains challenging due to yield limitations, complex purification workflows, and the need for standardized potency assays, whereas synthetic carriers benefit from more established GMP-compatible manufacturing frameworks [12,76,81,97,101]. Regulatory pathways for EV therapeutics may also be more complex, as they intersect biologics, cell-derived products, and advanced therapy classifications, necessitating rigorous characterization of composition, biodistribution, and long-term safety [76,77,98,99,102]. Thus, while EVs provide unmatched biological integration and multi-component metabolic signaling capacity, synthetic nanoparticles retain advantages in scalability, manufacturing reproducibility, and regulatory clarity [13,47,82,100]. A balanced assessment suggests that EV-based delivery is particularly advantageous when biological compatibility and coordinated metabolic modulation are priorities, whereas synthetic systems may be preferable when standardized, high-yield cargo delivery is required [12,13,20,82].

## 5. CRISPR-EV Synergy: Mechanisms and Data

**Figure 2:**
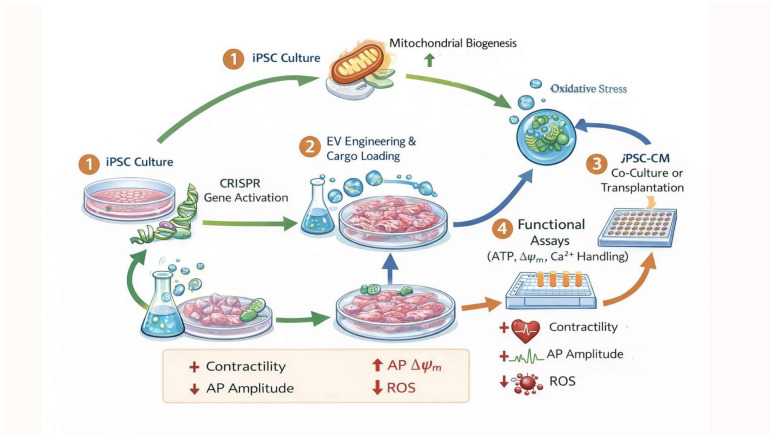
Integrated CRISPR–EV workflow for metabolic maturation of iPSC-derived cardiomyocytes. Stepwise schematic illustrating precision metabolic engineering: (1) CRISPR-mediated activation of mitochondrial biogenesis and oxidative metabolism regulators; (2) engineering of extracellular vesicles with metabolic and redox-modulating cargo; (3) delivery to iPSC-CMs followed by functional bioenergetic assays (ATP production, mitochondrial membrane potential, calcium handling); (4) endpoint quantification of contractility, electrophysiological maturation, and oxidative stress reduction. Arrows indicate directionality of metabolic change, with increased mitochondrial biogenesis and ATP production and corresponding reductions in oxidative stress and ROS burden [4,6,12,44]. Arrows indicate the direction of metabolic changes: green arrows = increase in mitochondrial biogenesis and ATP production; blue arrows = decrease in ROS and oxidative stress. Numbered steps correspond to sequential experimental phases described in Section 5.

Coordinating genomic engineering with extracellular metabolic modulation represents a cutting-edge strategy in precision cardiometabolic reprogramming [6,11,12,13]. Individually, CRISPR-based editing defines intracellular metabolic landscapes by altering gene regulation of mitochondrial biogenesis and substrate utilization [6,11,72]; extracellular vesicles (EVs) serve as endogenous metabolic nanocarriers that adjust recipient cell energetics and organelle dynamics [12,13,16,48]. Their synergy harmonizes genetic and metabolic layers of control, yielding quantitative improvements in mitochondrial structure, calcium homeostasis, electrophysiological stability, and bioenergetic performance unmatched by either modality alone [6,10,11,12,13,48]. Figure 2 outlines the integrated CRISPR-EV metabolic engineering pipeline, illustrating how sequential genetic programming and extracellular modulation converge to produce quantifiable functional maturation of iPSC-CMs [6,11,12,13]. The numbered steps in Figure 2 correspond to sequential experimental phases, from genomic programming to extracellular metabolic reinforcement and functional endpoint analysis.

### 5.1. Complementary Actions: Gene vs. Extracellular Modulation

CRISPR-guided systems offer unparalleled control over transcriptional networks, enabling targeted activation (CRISPRa) of mitochondrial biogenesis regulators (e.g., PGC-1α, ERRα/β) or repression (CRISPRi) of deleterious metabolic signals [58,61,62,72]. This genomic precision fundamentally shifts metabolic flux by enhancing oxidative phosphorylation capacity, electron transport chain (ETC) assembly, and mitochondrial network expansion [6,11,25].

In contrast, EVs influence metabolic phenotype post-transcriptionally by delivering metabolic enzymes, regulatory miRNAs, and even mitochondrial substructures into recipient cardiomyocytes [12,14,20,21,84]. EV cargo modulates key mitochondrial dynamics proteins (e.g., mitofusins MFN1/2 and DRP1) [12,40,41] to rebalance fusion/fission equilibrium and restore membrane potential (Δψm) and ATP synthesis efficiency [13,16,48]. The genomic alterations driven by CRISPR complement EV-mediated biochemical effectors by establishing a pro-oxidative transcriptional environment into which EV cargo can be more functionally integrated, achieving amplified metabolic remodeling [6,11,13].

This multilayered interaction integrates transcriptional programming with extracellular cargo delivery, creating a robust feedback loop in which engineered EVs reinforce CRISPR-induced metabolic gene networks and CRISPR modulation amplifies the responsiveness of recipient cells to EV cues.

Importantly, EV cargo composition can be deliberately engineered to achieve targeted mitochondrial modulation [12,14,82]. Donor cell metabolic preconditioning (e.g., hypoxia exposure, oxidative stress adaptation, or fatty acid enrichment) alters intracellular signaling pathways and selectively enriches EVs with cardioprotective miRNAs, antioxidant enzymes, and mitochondrial regulatory proteins reflective of the induced metabolic state [42,85,90,91]. Transient transfection of donor cells with miRNA mimics, plasmids encoding mitochondrial factors, or CRISPR-based transcriptional activators further enables programmable enrichment of defined cargo within secreted EVs [11,70,71,82]. Post-isolation loading strategies—including electroporation, membrane permeabilization, and lipid-based encapsulation—permit incorporation of synthetic RNAs, recombinant proteins, or small-molecule metabolic modulators directly into EVs [13,82,93]. In addition, surface engineering approaches (e.g., genetic fusion of targeting peptides to EV membrane proteins such as CD63 or Lamp2b) enhance tissue specificity and uptake efficiency [23,81,82]. CRISPR-mediated editing of EV-producing cells can also stably reprogram cargo packaging pathways, biasing vesicles toward enrichment of mitochondrial biogenesis regulators, redox enzymes, or metabolic transcription factors [11,63,66,103]. Collectively, these strategies provide mechanistic control over EV cargo composition, enabling precision tuning of mitochondrial dynamics, oxidative phosphorylation capacity, and bioenergetic resilience in recipient cardiomyocytes [12,38,40,47].

### 5.2. Enhanced Mitochondrial Fusion/Fission Balance

Mitochondrial dynamics are central to cardiomyocyte function: balanced fusion enhances respiratory efficiency and calcium handling [37,38,40], while unchecked fission fosters fragmentation [39,41,43], ROS accumulation [39,41,43], and apoptosis [39,41,43]. EV cargo modulates these pathways by influencing phosphorylation states of DRP1 (reducing fission) and elevating expression of fusion mediators MFN1, MFN2, and OPA1 [12]. EVs also carry signaling miRNAs that downregulate fission-promoting factors, facilitating networked mitochondrial architectures that optimize oxidative performance [14,84].

When paired with CRISPR modulation that upregulates TFAM, PGC-1α, and ERR-regulated mitochondrial genes, the combined effect is a mitochondrial network that is both transcriptionally primed and structurally stabilized [11,49,50,51,52,53,54]. Bioenergetic consequences include improved electron flux through complexes I–IV [37,39], reduced ROS emission, and increased mitochondrial DNA replication—outcomes critical for long-term energetic resilience [31,39,42].

### 5.3. Calcium Handling and Electrophysiologic Improvements

Mitochondrial and metabolic integrity directly influences cardiomyocyte electrophysiology and calcium homeostasis. Restored Δψm enhances the capacity for calcium uptake by the mitochondrial calcium uniporter (MCU), improving excitation–contraction coupling and mitigating arrhythmogenic diastolic calcium leaks [38,40]. EV-mediated restoration of mitochondrial membrane potential and redox balance supports more stable intracellular Ca^2+^ transients and lowers pro-arrhythmic triggers [13,16].

CRISPR-based editing of calcium-handling genes (e.g., ion channels and ryanodine receptors) in concert with EV-delivered metabolic modulators further stabilizes cardiac electrophysiology by reducing calcium overload and improving rhythmicity [10,18]. CRISPRi modulation of pathological pathways also prevents maladaptive calcium cycling that can emerge under high metabolic stress, bridging electrophysiological stability with metabolic robustness [10,72].

### 5.4. Quantitative Metrics Across Studies

Across preclinical models, the synergistic combination of CRISPR and EV metabolic modulation yields measurable improvements in key bioenergetic indicators:

- ATP Production: EV transfers of mitochondrial cargo enhance ATP synthesis, while CRISPR-mediated upregulation of ETC and biogenesis genes increases ATP output synergistically in recipient cardiomyocytes [45]. In mitochondrial-rich EV (M-EV) studies, treatment restored intracellular ATP levels to near-physiological levels within hours after administration [11].
- Oxidative Respiration: EV-induced mitochondrial fusion [12] and CRISPR-enhanced OXPHOS gene networks significantly improve basal and maximal oxygen consumption rates (OCR) [11], quantifiable through Seahorse assays and other bioenergetic profiling platforms [6,17].
- Mitochondrial Health: Quantitative assessments show increased mtDNA copy number, elevated complex I–IV activity, and reduced ROS accumulation when EV and CRISPR strategies are combined, outperforming either intervention alone [8,16,28].
- Functional Outcomes: In cardiac injury models, these interventions synergize to improve contractile performance, reduce infarct size, and stabilize calcium transients, reflecting integrated improvements in metabolism-linked cardiac physiology [10,17].

The cumulative functional advantages conferred by CRISPR-EV combinatorial strategies across preclinical systems are consolidated in Table 3.

### 5.5. Synthesis and Future Perspectives

CRISPR-EV synergy represents a holistic metabolic engineering frontier, merging genome-level programming with extracellular metabolic modulation to reconfigure cardiomyocyte bioenergetics and physiology. By addressing both intracellular genomic circuits and extracellular signaling networks, this approach enables a systems-level transformation—enhancing mitochondrial dynamics, calcium homeostasis, and electrophysiological stability with precise quantitative control [6,7,25].

The integration of CRISPR gene editing and engineered EV delivery platforms holds exceptional promise for translational cardiac therapies, bridging preclinical efficacy with scalable precision metabolic modulation [1,8,77]. Achieving standardized quantitative assays, rigorous mechanistic validation [64,65,66,67,68,77,78], and optimized delivery systems [82,83] will be essential next steps in elevating this synergistic paradigm toward clinical application.

## 6. Biomolecular Biomarkers and Quantitative Platforms

A systematic understanding of cardiomyocyte metabolic reprogramming hinges upon quantitative biomarkers that faithfully reflect cellular energetics, redox dynamics, and mitochondrial performance. Integrating NAD^+^/NADH ratios, ATP turnover measurements, global metabolomic signatures, and high-resolution respirometry not only enriches mechanistic insight but also provides scalable metrics for comparative evaluation across engineered cell systems. Coupled with advanced bioinformatic integration and predictive modeling, these platforms form an analytical framework capable of driving precision metabolic engineering strategies forward [1,2,6,7,23,24].

### 6.1. NAD^+^/NADH Ratio, ATP Turnover, and Metabolomics

The NAD^+^/NADH ratio is a central redox biomarker linking metabolic pathways to energy state and cellular stress responses. NAD^+^ acts as an electron acceptor for glycolysis, TCA cycle enzymes, and oxidative phosphorylation, while NADH donates electrons to the electron transport chain (ETC) at complex I [6,7,34,41,42]. A high NAD^+^/NADH ratio typically correlates with efficient oxidative metabolism and robust mitochondrial respiration, whereas elevations in NADH reflect a shift toward glycolytic reliance and reduced ETC flux [6,7,34,41]. Perturbations in this ratio influence not only ATP synthesis but also sirtuin activity and protein acetylation states that govern transcriptional control of metabolic genes [6,34,42]. In iPSC-derived cardiomyocyte (iPSC-CM) models of mitochondrial dysfunction, alterations in NAD^+^/NADH directly associate with impaired calcium cycling and arrhythmic phenotypes, underscoring its utility as a mechanistic and functional biomarker [10,32].

Quantifying ATP turnover rates provides direct evaluation of bioenergetic capacity and substrate utilization efficiencies [6,23,24,30]. Methods such as those developed with the Seahorse XF Analyzer allow simultaneous assessment of ATP production attributable to oxidative phosphorylation versus glycolysis [23,24,30]. By calculating ATP production rates from oxygen consumption and extracellular acidification data, researchers can distinguish the contributions of metabolic pathways under basal and stressed conditions, facilitating high-resolution comparisons across genetic or engineered perturbations.

Metabolomics offers a comprehensive snapshot of small-molecule metabolites within key pathways including glycolysis, TCA cycle intermediates, lipid metabolism, and amino acid flux [5,17,34]. Through high-resolution mass spectrometry and capillary electrophoresis-based platforms, hundreds of metabolites can be annotated and compared across conditions, revealing signatures of metabolic maturation or disease states. For example, principal component analysis of iPSC-CM metabolomes can uncover discrete clusters representing metabolic states, while ratios such as glutathione redox balance (GSH/GSSG) further indicate oxidative stress and energy perturbation [42,43].

Together, NAD^+^/NADH, ATP dynamics, and metabolite profiling comprise a multidimensional biomarker signature that quantifies metabolic performance and informs engineering strategies.

### 6.2. Seahorse XF, High-Resolution Respirometry

The Seahorse XF extracellular flux analyzer has emerged as a cornerstone in metabolic phenotyping, enabling real-time measurement of oxygen consumption rate (OCR) and extracellular acidification rate (ECAR) in living cells. OCR is a direct proxy for mitochondrial respiration, reflecting basal respiration, ATP-linked OCR, maximal respiratory capacity, and spare respiratory capacity [24,25,30]. ECAR predominantly reflects glycolytic activity through lactate production, providing a complementary dimension to oxidative metrics [24,25,30]. Seahorse assays also support perturbation protocols using inhibitors like oligomycin, FCCP, and rotenone/antimycin to dissect specific components of respiratory control [24,25].

In iPSC-CM models, Seahorse profiling reveals how metabolic maturation strategies—whether it be genetic editing or EV-mediated modulation—shift the balance between glycolysis and oxidative metabolism [5,6,17,25]. High baseline “proton leak” respiration seen in immature cells diminishes following engineering, with concomitant increases in ATP-linked respiration and spare capacity, indicating improved coupling and functional mitochondria [6,35].

High-resolution respirometry platforms such as Clark-type electrodes and Oroboros O2k complement Seahorse data by offering mechanistic specificity and kinetic resolution of mitochondrial states [6,35,41]. These platforms enable analysis of isolated mitochondria and intact cells to tease apart substrate-dependent respiration and proton leak dynamics across metabolic conditions.

### 6.3. Bioinformatic Integration and Predictive Modeling

The wealth of data generated from metabolomics, NAD^+^/NADH assays, ATP turnover measurements, and respirometry necessitates sophisticated bioinformatic integration frameworks capable of multi-omics synthesis [2,9,24]. Integration approaches—from multi-omics data harmonization to constraint-based metabolic modeling—enable researchers to map metabolic networks, identify latent relationships between genes and metabolites, and predict the systemic effects of perturbations [2,6,24,25]. Multi-omics integration capitalizes on shared patterns across transcriptomic, proteomic, and metabolomic layers to reconstruct functional modules governing metabolic state.

Predictive modeling frameworks such as flux balance analysis (FBA) use genome-scale metabolic reconstructions constrained by empirical data to simulate metabolic flux distributions under varying conditions. These models leverage stoichiometric representations of metabolism to predict how interventions (e.g., genetic edits or EV cargo modulation) alter pathway usage, ATP yield, and redox balances [6,24,25]. This computational layer facilitates hypothesis generation and experimental prioritization, dramatically accelerating iterative design cycles.

Advanced machine learning and differential equation–based approaches further enhance predictive abilities, enabling temporal simulation of pathway dynamics from time-series omics datasets [2,6,9]. These tools can reveal latent interactions and forecast metabolic responses to engineered perturbations with high fidelity, offering a pathway to data-driven optimization of metabolic engineering strategies [2,6,9,25].

In conclusion, quantitative metabolic biomarkers and analytical platforms form the empirical foundation of precision metabolic engineering in cardiomyocytes. Integration of redox ratios, ATP kinetics, global metabolomics, and real-time respirometry data—coupled with bioinformatic and predictive modeling—enables high-resolution characterization and dynamic optimization of engineered cellular metabolism, positioning engineered iPSC-CMs for translational success [1,2,5,6,7,17,24,25].

## 7. Translational Framework

Translating precision metabolic engineering of iPSC-derived cardiomyocytes (iPSC-CMs) and engineered extracellular vesicles (EVs) into clinical reality requires a rigorous and systematic framework that integrates preclinical efficacy and safety data, scalable manufacturing approaches, and alignment with regulatory landscapes governing advanced biologics and regenerative therapies [1,2,4,8,15].

### 7.1. Preclinical Efficacy and Safety Data

Preclinical models serve as the foundational evidence base for advancing regenerative cardiometabolic therapies toward human trials [8,24,28]. Analyses of iPSC-CM therapy in ischemic heart disease animal models demonstrate measurable improvements in cardiac function. A meta-analysis across 51 studies with over 1000 animals showed that iPSC-CM transplantation significantly improved left ventricular ejection fraction (LVEF) by ~8.2% compared with controls, while also minimizing mortality and arrhythmia risk, suggesting a favorable safety profile and functional benefit in vivo [96].

In more complex preclinical systems, clinical-grade iPSC-CM patches have been evaluated for both safety and efficacy. In porcine models of myocardial infarction, implantation of iPSC-CM patches enhanced cardiac function and angiogenesis without observable tumor formation or lethal arrhythmias [8,28]. Systematic genomic and exome sequencing confirmed the absence of deleterious mutations, while general toxicity assays showed no adverse systemic effects [11,19,97].

Critically, these studies demonstrate not only functional improvement (contractility, perfusion) but also robust safety endpoints—no tumorigenicity, stable electrophysiology, and minimal immune-mediated responses—when cells are purified and quality-controlled [10,19,24]. They provide quantitative benchmarks (e.g., LVEF improvements, fibrosis reduction) vital for designing early clinical trials [8,24,28].

For engineered EVs, preclinical evidence illustrates reduction in inflammation, diminished cardiomyocyte apoptosis, smaller infarct sizes, and improved cardiac performance through delivery of cardioprotective cargo [4,13,16,20,21,48]. These results support the deployment of EVs as cell-free biologics with lower immunogenicity and scalable delivery characteristics.

Despite positive signals, heterogeneity in experimental design and outcome measurement across studies emphasizes the need for standardized preclinical efficacy and safety protocols to harmonize endpoints such as functional cardiac output, arrhythmia incidence, and long-term tissue integration [8,10,23,24,48].

### 7.2. Cell Therapy Manufacturing Scalability

A central bottleneck in translational cardiometabolic therapies is the scalable and reproducible manufacturing of iPSC-CMs and engineered EVs under Good Manufacturing Practice (GMP) conditions [1,2,8,15,16]. Traditional 2D culture systems [1,2,8], while widely used in research, are labor-intensive and lack the control and throughput required for clinical production. Emerging suspension culture platforms and bioreactor systems enable higher cell yields with consistent culture parameters, addressing important aspects of quality, homogeneity, and batch-to-batch reproducibility [1,2,3].

Scalability challenges in iPSC platforms include variations in pluripotency maintenance, differentiation efficiency, and genetic stability during expansion [2,3,5,6]. Rigorous quality control—encompassing genomic integrity checks, phenotype validation, and contaminant screening—is essential to ensure safety and functional consistency in therapeutic cell products [11,19,63].

Automated and closed-system bioprocessing technologies further reduce manual error, minimize contamination risk, and support design-of-experiments (DoE) approaches for process optimization [2,15]. Implementation of xeno-free culture environments and recombinant extracellular matrices augments clinical compatibility and reduces risks associated with undefined components [2,15,26].

For engineered EV therapies, scalability entails robust EV isolation, cargo definition, and nanoparticle characterization, with optimized upstream production and downstream purification methods that maintain functionality while enabling reproducible pharmacologic profiles [12,14,16,21,23].

Integration of process analytical technologies (PAT) and real-time monitoring systems enhances manufacturing control and ensures product quality attributes align with regulatory expectations for cell and gene therapy products [2,15,16,98].

### 7.3. Regulatory Landscape

Navigating the regulatory environment is pivotal for successful clinical translation of iPSC-CM and engineered EV therapies [76,100]. In the United States, the Food and Drug Administration (FDA) classifies iPSC-derived products as biologics under Section 351 of the Public Health Service Act when they involve more than minimal manipulation or non-homologous use and require an Investigational New Drug (IND) application prior to human trials. Detailed chemistry, manufacturing, and control (CMC) data outlining identity, purity, potency, and safety are mandatory [76,100].

Regulatory agencies in Europe (European Medicines Agency, EMA) [101] and Japan (Pharmaceuticals and Medical Devices Agency, PMDA) [102] have analogous frameworks that emphasize Good Manufacturing Practice (GMP) compliance, quality risk management, and rigorous characterization of therapeutic products. Differences across regions necessitate harmonized approaches to quality standards and trial design to support global deployment [76,100,101,102].

Special designations such as FDA’s Regenerative Medicine Advanced Therapy (RMAT) status [104,105] can accelerate development pathways for therapies addressing serious conditions, but also require robust post-marketing safety plans and risk evaluation mitigation strategies.

### 7.4. Summary of Translational Imperatives

A translational framework for precision cardiac regenerative therapies must integrate quantitative preclinical efficacy and safety data, scalable, GMP-compliant manufacturing processes, and a nuanced understanding of multinational regulatory requirements. Harmonizing these elements enables the design of rigorous clinical trials with well-defined endpoints, supports regulatory submissions with strong quality and safety evidence, and accelerates the transition from laboratory innovation to therapeutic impact for patients with cardiovascular disease [1,2,4,8,15,16,24].

## 8. Future Directions and Challenges

As precision metabolic engineering, CRISPR technologies, and extracellular vesicle (EV)-based therapies advance toward clinical application, several critical translational hurdles and emerging opportunities remain. This chapter systematically examines three cornerstone challenges: (1) in vivo maturation and engraftment of engineered iPSC-derived cardiomyocytes (iPSC-CMs), (2) immune modulation via EVs, and (3) personalized metabolic editing strategies. Integrating quantitative biomolecular requirements with translational imperatives is essential to bridge preclinical promise and clinical reality.

### 8.1. In Vivo Maturation and Engraftment Hurdles

Despite substantial improvements in in vitro metabolic and structural maturation of iPSC-CMs, achieving robust in vivo performance remains a major challenge. In large animal models, metabolically optimized iPSC-CMs still show significant functional deficits compared with native cells—including reduced contractile force (up to ~42%) [4,6] and prolonged action potential duration [4,6]—leading to arrhythmogenic risk [1,2,10] and biomechanical integration issues post-implantation. These functional discrepancies suggest that metabolic reprogramming alone may not suffice for durable in vivo integration. Strategies that combine metabolic tuning with biomechanical and electrophysiological cues are under investigation. For example, electrical pacing, mechanical loading, and engineered scaffolds enhance sarcomeric alignment, calcium cycling, and mitochondrial function, which are crucial for physiological maturation in situ. However, effective, long-term engraftment with synchronized excitation–contraction coupling and immune tolerance remains elusive, underscoring the need for integrated multimodal maturation strategies.

In the longest prospective non-human primate study reported to date, autologous iPSC-CMs showed stable engraftment and evidence of maturation for over 6–12 months without immunosuppression, in contrast to allogeneic cells that were rejected within weeks [102]. This work emphasizes the importance of host–cell compatibility and immune regulation in fostering durable graft survival and functional integration.

Scaffold-based tissue engineering also shows promise; decellularized extracellular matrix (ECM) and anisotropic cardiac-specific scaffolds support iPSC-CM maturation and functional integration when transplanted in rodent myocardial infarction models, improving ejection fraction and reducing maladaptive remodeling.

Despite these advances, long-term retention rates (<10%) and variable functional coupling with host myocardium highlight the need for further refinement in design and delivery [106].

### 8.2. Immune Modulation via EVs

EVs possess inherent immunomodulatory potential that can be harnessed to shape the post-transplantation environment toward tolerance and repair. Stem cell-derived EVs, including those from mesenchymal stromal cells (MSCs) and iPSC derivatives, carry miRNAs and proteins that induce immune cell phenotypes supportive of regenerative processes. For example, EV cargo such as miR-181b, miR-182, and miR-146a has been shown to promote M2 macrophage polarization, suppress pro-inflammatory cytokines (TNF-α, IL-6), and enhance anti-inflammatory signaling in preclinical models, thereby reducing deleterious inflammation and creating a more permissive niche for tissue repair.

Beyond macrophage modulation, EVs influence T cell function and other adaptive immune components, producing a nuanced immunoregulatory effect that may be critical for reducing graft rejection without chronic immunosuppression. Although exosomes and microvesicles show potent immunoregulatory activity, their effects vary depending on source cell type, culture conditions, and administration timing, necessitating careful optimization for reproducible therapeutic outcomes.

Critical challenges include rapid clearance by macrophages and off-target biodistribution that limit EV accumulation at sites of injury. Addressing these issues through surface modification, targeting ligands, and controlled release systems will be necessary to maximize therapeutic utility.

### 8.3. Personalized Metabolic Editing Strategies

The increasing precision of genetic editing tools enables patient-specific metabolic interventions that extend beyond conventional CRISPR/Cas9 approaches. Prime editing (PE), for example, offers “search-and-replace” genome modification that corrects point mutations and indels without creating double-strand breaks [103,106,107], potentially reducing off-target risks and enabling more tailored metabolic edits in cardiomyocytes. While PE has demonstrated high precision and therapeutic potential in disease modeling, efficient in vivo delivery to cardiac tissue remains a significant barrier.

Personalized editing strategies could also integrate multi-omics patient data with high-throughput phenotyping to design individualized intervention profiles that optimize metabolic pathways for regenerative outcomes. Combining genome editing with patient-derived iPSC-CM models allows in vitro assessment of metabolic gene function and pharmacologic responses, enabling tailored metabolic engineering that reflects genetic background and disease phenotype.

Realizing such personalized approaches will depend on enhancing delivery systems (e.g., viral and non-viral vectors) and regulatory frameworks that accommodate individual genomic edits while ensuring safety, minimal immunogenicity, and durable efficacy. Additionally, strategies that combine transcriptomic profiling, metabolic flux analysis, and computational modeling may guide optimal editing targets for each patient.

### 8.4. Outlook

Addressing in vivo maturation and engraftment, immune modulation, and personalized editing strategies will be pivotal in advancing cardiometabolic regenerative therapies. Progress in tissue engineering, EV immunomodulation, and precise metabolic editing promises a next generation of targeted, patient-specific interventions capable of durable functional recovery in cardiovascular disease. Continued integration of quantitative biomolecular data, scalable delivery platforms, and immune-informed design will be essential to translate bench-top innovations into transformative clinical therapies.

## 9. Conclusions

The convergence of CRISPR-guided metabolic engineering and extracellular vesicle (EV)-mediated modulation represents a transformative platform for precision cardiac regeneration. Across the preceding chapters, we have systematically examined the molecular, cellular, and translational dimensions of this synergistic strategy, highlighting its potential to overcome longstanding limitations in iPSC-derived cardiomyocyte (iPSC-CM) therapy. By simultaneously targeting intracellular metabolic circuits and intercellular signaling networks, the CRISPR-EV paradigm enables coordinated enhancement of mitochondrial efficiency, substrate utilization, and energetic flexibility, addressing both intrinsic cellular immaturity and extrinsic microenvironmental constraints.

CRISPR-based interventions, including transcriptional activation/inhibition (CRISPRa/i), base editing, and prime editing, facilitate precise modulation of key metabolic regulators such as PGC-1α, TFAM, ERRα/β, and PPAR pathways. These modifications augment mitochondrial biogenesis, optimize oxidative phosphorylation, and improve ATP kinetics in iPSC-CMs, achieving functional maturation metrics closer to adult cardiomyocytes. Complementarily, engineered EVs deliver metabolic enzymes, miRNAs, and lipid modulators that refine recipient cell energetics, reduce reactive oxygen species accumulation, and stabilize calcium handling, thereby enhancing electrophysiologic performance and contractile efficiency. The integration of these approaches produces quantitative gains in mitochondrial membrane potential, NAD^+^/NADH balance, oxygen consumption rates, and ATP turnover, with preclinical studies demonstrating improvements in contraction force, rhythmicity, and tissue-level metabolic coordination.

This systematic framework offers a revolutionary trajectory toward precision cardiac regeneration, wherein patient-specific metabolic states can be iteratively modeled, optimized, and implemented using iPSC-CM platforms. Personalized strategies—guided by high-resolution respirometry, Seahorse XF assays, metabolomics, and multi-omics bioinformatic modeling—enable targeted interventions tailored to the unique energetic profiles of individual patients. In parallel, EV-mediated immune modulation creates a permissive microenvironment for engraftment, mitigating inflammatory responses and enhancing long-term survival of transplanted cardiomyocytes.

From a translational perspective, the CRISPR-EV synergy addresses critical bottlenecks in current regenerative paradigms: the metabolic immaturity of iPSC-CMs, suboptimal mitochondrial dynamics, arrhythmogenic risk, and limited systemic integration. By coupling gene-level precision editing with network-level intercellular modulation, this approach establishes a holistic platform capable of restoring energetic homeostasis, structural functionality, and electrophysiologic fidelity.

In conclusion, the CRISPR-EV strategy transcends conventional regenerative frameworks by providing a multi-scale, biomolecularly informed, and quantitatively validated methodology for cardiac repair. It embodies a paradigm shift from structural replacement toward systemic metabolic optimization, offering a scalable, reproducible, and precision-oriented pathway for next-generation cardiac regenerative medicine. Future clinical translation will depend on harmonizing manufacturing scalability, safety metrics, immune compatibility, and personalized editing protocols, establishing this dual-modality platform as a foundational pillar for precision cardiac regeneration.

## Figures and Tables

**Table 1:** Metabolic Profile Comparison of iPSC-CMs vs. Adult Cardiomyocytes. Metabolic profile comparison between iPSC-derived cardiomyocytes and adult human cardiomyocytes. This table contrasts key bioenergetic parameters including dominant ATP source, oxygen consumption rate (OCR), mitochondrial morphology, fatty acid oxidation capacity, NAD^+^/NADH balance, and reactive oxygen species (ROS) burden handling. ROS levels were quantified using fluorescence-based probes and expressed as relative fluorescence intensity normalized to adult cardiomyocytes. The comparison establishes the baseline metabolic immaturity of iPSC-CMs and defines priority nodes for CRISPR- and extracellular vesicle-based metabolic interventions.

Feature	iPSC-CMs	Adult CM	% Relative Deviation from Adult CM	Therapeutic Target
ATP production (pmol/cell/min)	45 ± 5 [25]	120 ± 10 [17,26]	−62%	PGC-1α, TFAM
Oxidative Phosphorylation contribution (%)	35 ± 7 [6]	80 ± 5 [6]	−56%	PPARδ, ERRα
Glycolytic reliance (%)	65 ± 6 [6,17,28,29,30]	20 ± 4 [6,30]	+225% (Pathological excess)	Metabolic shift via EV miRs
Mitochondrial DNA (mtDNA) density (#/cell)	1000–4000 [22,31,32,33,34]	4000–6000 [31,32,33,34,35]	−50%	CRISPR-mediated biogenesis
ROS burden (fold vs. adult)	3.0-fold ↑ [6]	1.0-fold ↑ [6]	+200% (Pathological Excess)	EV antioxidant cargo

Symbol “#” means number of mtDNA density per cell. Symbol “↑” means an upward increase.

**Table 2:** Engineered EV Cargo for Metabolic Rescue. Engineered extracellular vesicle cargo and associated metabolic outcomes in cardiomyocyte models. The table catalogs EV-delivered miRNAs, proteins, lipids, and mitochondrial components alongside their documented effects on ATP production, mitochondrial membrane potential, oxidative stress reduction, and substrate flexibility. Data are derived from recent preclinical studies evaluating EV-mediated metabolic rescue in cardiac injury and iPSC-CM maturation models.

EV Cargo	Molecular Function	Target	Functional Outcome	% Change (Preclinical)
miR-210 [91]	Hypoxia adaptation	HIF1α pathway	↑ OXPHOS	+35–45%
miR-199a [92]	Energy metabolism	PGC-1α	↑ ATP production	+28–40%
CRLS1 [93]	Cardiolipin synthesis	Mitochondrial inner membrane	↑ Δψm	+30–50%
SOD2 protein *(#/cell)* [94]	ROS scavenging	Mitochondria	↓ ROS	−45%
Citrate synthase mRNA [95]	TCA flux	Krebs cycle	↑ basal respiration	*EV* + 20–35%

Symbol “#” means number of SOD2 protein per cell. Symbol “↑” means an upward increase, “↓” means an downward increase.

**Table 3:** Quantitative Functional Outcomes of CRISPR-EV Metabolic Reprogramming. Quantitative functional outcomes of CRISPR-EV-mediated metabolic reprogramming. This table summarizes key preclinical performance metrics—including ATP output, oxygen consumption rate, mitochondrial DNA copy number, ROS reduction, calcium transient stability, and contractile enhancement—demonstrating the superior efficacy of combined CRISPR and EV strategies compared with single-modality interventions.

Functional Parameter	Baseline iPSC-CMs	CRISPR Only	EV Only	CRISPR + EV	% Improvement vs. Baseline
ATP (pmol/cell/min)	45 ± 5	85 ± 7	70 ± 6	105 ± 8	+133%
Maximal respiration (OCR, pmol/min)	30 ± 3	60 ± 5	50 ± 4	75 ± 6	+150%
Calcium transient amplitude (ΔF/F_0_)	0.8 ± 0.1	1.0 ± 0.1	0.95 ± 0.1	1.1 ± 0.1	+37.5%
ROS (µM)	15 ± 2	12 ± 1	10 ± 1	8 ± 0.8	−46%
Mitochondrial density (#/cell)	200 ± 30	500 ± 40	400 ± 35	700 ± 50	+250%

Symbol “#” means number of mitochondrial density per cell.

## Data Availability

No new data were created or analyzed in this study.
